# A Highly Sensitive Impedimetric DNA Biosensor Based on Hollow Silica Microspheres for Label-Free Determination of *E. coli*

**DOI:** 10.3390/s20051279

**Published:** 2020-02-26

**Authors:** Eda Yuhana Ariffin, Lee Yook Heng, Ling Ling Tan, Nurul Huda Abd Karim, Siti Aishah Hasbullah

**Affiliations:** 1 Department of Chemical Sciences, Faculty of Science and Technology, Universiti Kebangsaan Malaysia (UKM), 43600 UKM Bangi, Selangor Darul Ehsan, Malaysia; edayuhana@ukm.edu.my (E.Y.A.); nurulhuda@ukm.edu.my (N.H.A.K.); aishah80@ukm.edu.my (S.A.H.); 2 Southeast Asia Disaster Prevention Research Initiative (SEADPRI-UKM), Institute for Environment and Development (LESTARI), Universiti Kebangsaan Malaysia, 43600 UKM Bangi, Selangor Darul Ehsan, Malaysia; lingling@ukm.edu.my

**Keywords:** DNA biosensor, *E. coli* detection, label-free DNA biosensor, impedimetric DNA sensor

## Abstract

A novel label-free electrochemical DNA biosensor was constructed for the determination of *Escherichia coli* bacteria in environmental water samples. The aminated DNA probe was immobilized onto hollow silica microspheres (HSMs) functionalized with 3-aminopropyltriethoxysilane and deposited onto a screen-printed electrode (SPE) carbon paste with supported gold nanoparticles (AuNPs). The biosensor was optimized for higher specificity and sensitivity. The label-free *E. coli* DNA biosensor exhibited a dynamic linear response range of 1 × 10^−10^ µM to 1 × 10^−5^ µM (R^2^ = 0.982), with a limit of detection at 1.95 × 10^−15^ µM, without a redox mediator. The sensitivity of the developed DNA biosensor was comparable to the non-complementary and single-base mismatched DNA. The DNA biosensor demonstrated a stable response up to 21 days of storage at 4 ℃ and pH 7. The DNA biosensor response was regenerable over three successive regeneration and rehybridization cycles.

## 1. Introduction

*Escherichia Coli* (*E. coli*) O157:H7 is an important foodborne and waterborne pathogen that can lead to cramps, bloody diarrhea, kidney failure, and other diseases in humans. This *E. coli* O157:H7 strain generates a strong and sturdy toxin that lead to severe illness [1]. In 1982, *E. coli* O157:H7 was first identified in contaminated hamburgers, causing disease [1]. However, some cases have been waterborne. In 1999, people became sick after drinking contaminated water in Washington county in New York and after swimming in contaminated water in Clark country, Washington. In 2014, traces of *E. coli* were found in water in Batu Ferringhi, Malaysia [2]. *E. coli* traces have also been found in Malaysia’s flooded north-east [3]. 

Electrochemical biosensors based on oxidation labels are widely used for the recognition of the *E. coli* DNA hybridization reaction. Oxidation labels function as hybridization markers that intercalate to double-stranded DNA and gives electrochemical or optical signals. Tiwari et al. [4] explored an electrochemical genosensor for *E. coli* detection based on graphene oxide, nickel ferrite and chitosan, with methylene blue as the redox indicator. Ariffin et al. [5] developed an electrochemical DNA biosensor for *E. coli* detection based on hollow silica spheres (HSMs), using an anthraquinone redox intercalator as the electroactive DNA hybridization label. However, hollowed silica spheres used as DNA immobilization sites have the potential to absorb this oxidation marker and distort the reading. This limitation can be overcome by using a label-free DNA biosensor [6]. 

The electrochemical label-free detection technique has shown excellent potential for DNA biosensors. This technique relies on conversion information from DNA base pair recognition into an electrical signal. It removes the requirement for additional modifications of biomolecules markers. This label-free detection technique is capable of detecting changes in the charge transfer process on the electrode surfaces [7]. The impedance technique is one of the alternatives that is used in designing label-free biosensors that is sensitive, quick, and cheap. This technique is constructed based on impedance system sampling as the interface changes. This change is intrinsic to biological recognition and requires no prerequisite condition (such as a marker or electroactivity in the molecule) in its measurement [8].

Impedance spectroscopy is an important technique for the analysis of electrochemical and electronic systems [9]. Electrochemical impedance spectroscopy (EIS) is a potent technique for DNA determination, as the resistance and capacitance values are the sensitive indicators of changes of the surface properties, which can be identified by modelling the electrochemical data [10]. Furthermore, EIS is a non-destructive technique that can provide time-dependent information about the ongoing process [10]. 

The primary key to developing a sensitive and selective biosensor relates to the surface, which facilitates implantation of the biological identification layer. For transducer material impedance measurement, the material needs to have a high conductivity level and low electron transfer resistivity on the surface. A high surface area for biomolecule immobilization is also essential to produce a functional biosensor [8].

Various studies have been conducted on producing hybridized label-free biosensors for *E. coli* detection. A label-free DNA aptamer-based impedance biosensor was developed for detecting *E. coli* outer membrane proteins (OMPs) [11]. Nurulasma et al. [12] developed a new label-free impedimetric DNA biosensor based on graphene nanosheets for the detection of *E. coli*. This method is selective, reproducible, and has a low detection limit, but the linear response is short. Brosel-Oliu [13] reported on an impedimetric aptasensor with a three-dimensional interdigitated electrode array transducer for selective *E. coli* with a low detection limit. Shen et al. [14] fabricated a label-free quartz crystal microbalance (QCM) immunosensor for detecting *E. coli* O157:H7 based on beacon immunomagnetic nanoparticles and colloidal gold. This method has high specificity and stability, but the method is complex and time consuming. It takes 4 h to detect *E. coli*.

Recently, impedance DNA biosensors have attracted scientists’ attention. Impedimetric biosensors for detection of dengue serotypes had been developed at a very low concentration by utilizing a hybrid composite of polyaniline and gold nanoparticles [15]. Deng and Toh [10] also reported on a nanoporous alumina-membrane-based impedance biosensor for dengue virus-specific oligonucleotide sequence detection. Fu et al. [16] fabricated a hybridized label-free DNA sensor based on gold nanoparticles and double-layered, two-dimensional 3-mercaptopropyltrimetoxilane inserted into a nanogold substrate.

Herein, we report a new rapid and sensitive aminated hollow silica microsphere (HSM)-supported, AuNP-modified, screen-printed electrode (SPE) for the identification of the *E. coli* DNA using electrochemical impedance spectroscopy. This label-free DNA biosensor can simplify the current biosensor detection process. The hybridization of the label-free DNA biosensor is determined based on the charge transfer resistance (R_CT_) principle on the electrode surface. It is noteworthy that the label-free *E. coli* DNA biosensor was developed without the usage of any redox mediators. 

## 2. Materials and Methods

### 2.1. Instrumentation

The impedance spectra were recorded in the frequency range of 100 to 1000 kHz (model PGSTAT 302N, Autolab, The Netherlands). The amplitude of the applied sine wave potential was 10 mV. The corresponding values of the real and imaginary parts of the impedance (Z’ and Z’’, respectively) were processed through the Frequency Response Analysis (FRA ) system software (Autolab, The Netherlands). The electrochemical system involved a three-electrode setup, containing screen-printed electrodes (SPE) modified with HSMs and gold nanoparticles (AuNPs), platinum electrodes, and Ag/ACl as a reference electrode. Electrochemical Impedance Spectroscopy (EIS ) measurements were performed in 0.05 M K-phosphate buffer at pH 7.

### 2.2. Chemicals

The (3-aminopropyl) triethoxysilane (APTES, 98%), glutaraldehyde, gold nanoparticles powder (AuNPs, <100 nm), potassium chloride (KCl), sodium chloride (NaCl), tetraethyl orthosilicate (TEOS, 98%), and ethanol were purchased from Fluka. Tween 20 surfactant and sodium fluoride (NaF, 98.5%) were supplied by R&M Chemicals and Merck, respectively. All aqueous solutions were prepared using MiliQ deionized water. The stock solution of the DNA probe was prepared in 0.05 M K-phosphate buffer (pH 7), while the complementary DNA (cDNA) solution was prepared in 0.05 M Na-phosphate buffer containing 0.1 M NaCl at pH 7. All synthetic DNA oligonucleotides were purchased from Sigma-Aldrich, including DNA probe sequences of *E. coli*, DNA probe AmC_3_(5’-GGTCCGCTTGCTCTCGC), complementary target sequences (5’-GCGAGAGCAAGCGGACC), and non-complementary target sequences (5’-CTAGTCGTATAGTAGGC). All probes were used by referring to Paniel and Baudart [17].

### 2.3. Synthesis of Hollow Silica Microspheres 

Aminated hollow silica microspheres (HSMs) were synthesized based on the report of Boissiere et al. [18], with some modifications. TEOS and Tween 20 were mixed at a molar ratio of 8:1 M/M (TEOS/Tween 20) and stirred for 10 min, followed by the addition of NaF into the mixture, and then stirred again for 3.5 h. The HSM solution was kept in a shaking water bath at 27 ℃ and the precipitate was sequentially washed with ethanol and deionized water by centrifugation of 4000 rpm for 15 min each time. The resultant HSMs were dried and calcined at 200 ℃ for 6 h and 620 ℃ for 6 h. Next, the synthesized HSMs were treated with 2 ml APTES and stirred overnight. The white slurry hollow silica sphere was air-dried for 24 h. 

### 2.4. Fabrication of Label-Free DNA Biosensor for E. coli Pathogen Detection

**Figure 1:**
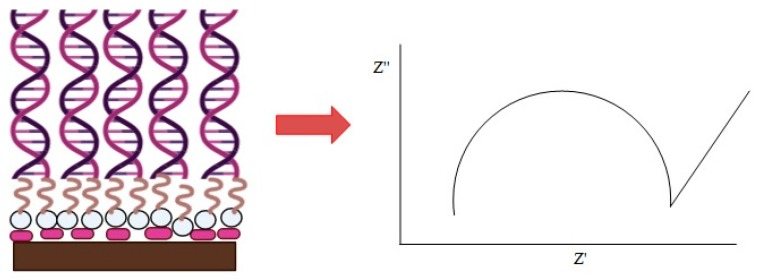
The fabrication of the label-free DNA biosensor, consisting of supported gold nanoparticles (AuNPs), hollow silica microspheres (HSMs), and the *E. coli* DNA probe for the detection of the *E. coli* pathogen.

AuNPs were dispersed in a solution that contained 30% ethanol and 70% deionized water. The carbon SPE was first modified with AuNPs by dropping AuNPs and air drying them at room temperature (27 ℃). Aminated hollow silica microspheres (HSMs) were dispersed in 95% ethanol and then dropped onto the AuNP-modified SPE (AuNPs-SPE). HSM/AuNPs/SPE was immersed in 8% glutaraldehyde for 1 hr followed by 1 µM DNA probe solution overnight to ensure the aminated DNA was covalently bonded to aminated HSMs via a glutaraldehyde crosslinking agent. The resulting DNA biosensor was carefully rinsed with 0.05 M K-phosphate buffer at pH 7 to remove the unbound DNA probe. DNA hybridization was performed by immersing the DNA biosensor in 300 µL of target DNA solution for 1 h at 27 °C. The DNA biosensor was then rinsed again with 0.05 M K-phosphate buffer at pH 7 to remove the excess cDNA residue. Figure 1 illustrates the fabrication of the label-free DNA biosensor. 

### 2.5. Analytical Performance of Label-Free DNA Biosensor

The linear response range and lower detection limit of the biosensor were examined for this study. The responses of the biosensors were measured three times in two batches of biosensors for a reproducibility study. For the selectivity behavior, a DNA biosensor was exposed to cDNA and non-cDNA solutions at two different concentrations. The lifetime of the DNA biosensor was determined by measuring a batch of biosensors on every specific day. 

### 2.6. Real Samples

The label-free *E. coli* DNA biosensor was verified and confirmed by standard plating method according to the FDA’s Bacteriological Analytical Manual for *E. coli* determination in water samples. Water samples were taken from different locations of the Langat River. All samples were autoclaved and filtered with filter paper (45 μm) to remove unnecessary large particles. The 100 mL samples were filtered again with a 100-grid membrane filter (pore size 0.2 μm) and poured onto two different kinds of agar, namely chromocult coliform agar and eosin methylene blue agar. All samples were incubated overnight for bacterial culture purposes. Biochemical tests, such as catalase, oxidase, and indole tests, were performed on *E. coli* bacteria that grew on the agars. *E. coli* ATCC25922 bacteria was used as a standard benchmark. For biosensor analysis, all water samples were heated at 80 ℃ and sonicated for 20 min to obtain the bacterial DNA [19,20]. The obtained DNA was hybridized with a label-free *E. coli* DNA biosensor and the signal was compared with the non-hybridized biosensor. All glassware was steriled.

## 3. Results and Discussion

### 3.1. Morphology and Characterization Studies of Hollow Silica Microspheres

**Figure 2:**
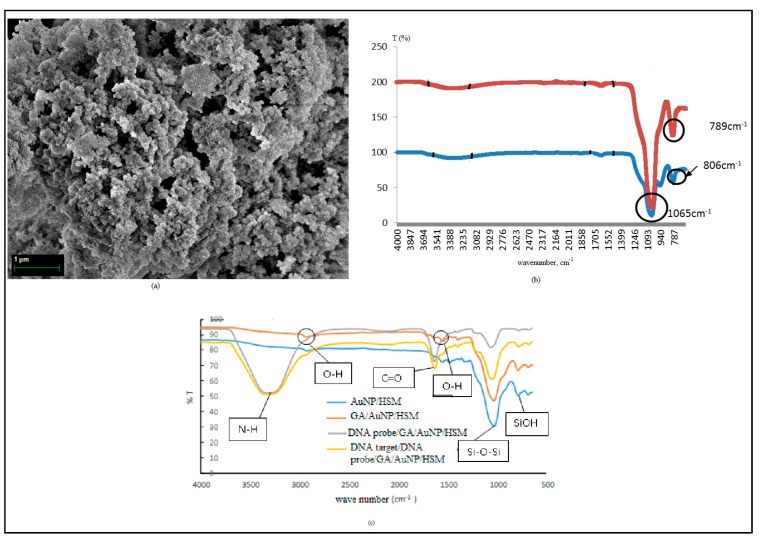
(**a**) FESEM micrograph of HSMs. (**b**) FTIR spectra of HSMs and (**c**) FTIR spectra of the DNA biosensor.

The results from the Field Emission Scanning Electron Microscope (FESEM) analysis (Figure 2a) show the morphology of the HSMs. The average physical pore size is approximately 150 nm. The HSMs increase the surface area for DNA immobilization and enhances the DNA biosensor sensitivity. The small size of the DNA is suitable for immobilization of the inner and outer shells of HSMs [21].

Infrared spectroscopy was used to characterize the features of HSMs before and after the calcination process (Figure 2b). The FTIR spectra of the HSMs before and after calcination show absorption bands at 1640 cm^−1^ and 3388 cm^−1^, corresponding to a bending vibration of the O–H group (1640 cm^−1^) and hydrogen bonding from the interaction with the silanol group (Si-OH) on the silica surface [22,23]. Before calcination, the absorption bands appeared at 806 cm^−1^ and 1065 cm^−1^, which are assigned to the siloxane symmetry group (Si-O-Si) and siloxane asymmetry streching vibration (Si-O-Si), respectively. The absorption band at 949 cm^−1^ (Si-OH) indicated the high concentration of the silanol group [23]. After calcination at 620 ℃ for 6 h, the absorption band at 1065 cm^−1^ (Si-O-Si asymmetric stretching) became sharper and the absorption band at 949 cm^−1^ (Si-OH bending vibration) disappearred [24] due to condensation of the silanol group, changing into the Si-O-Si network [25]. Si-O-Si symmetric vibration stretching shifted from 806 cm^−1^ to 789 cm^−1^, and the intensity of the absorption band increased due to the formation of the SiO_2_ network [26]. Two strong bands at around 1065 and 789 cm^−1^ indicated Si-O-Si vibration of the hollow silica, demonstrating that calcination stimulated complete oxidation of the template [27,28]. 

Figure 2c shows the FTIR spectra for the DNA biosensor. Glutaraldehyde (GA) is a crosslinking agent and was used to attach biomolecules, such as DNA or enzymes, onto HSMs. After the aminated DNA probe was immobilized onto GA/HSM/AuNP, a broad and clear FTIR adsorption band was formed in between wavenumber 2970 and 3690 cm^−1^. Broad and strong peaks at wavenumber ~3310 cm^−1^ refer to the N-H asymmetry stretch. A sharp peak at ~1655 cm^−1^ refers to the C=O stretching of the primary amine group, which clearly shows the presence of aminated the DNA probe immobilized onto HSMs [22]. The aminated DNA probe immobilized sucessfully onto HSMs, even though the HSMs were dropped onto AuNPs.

### 3.2. Electrochemical Biosensor Characterization in Na-Phosphate Buffer

**Figure 3:**
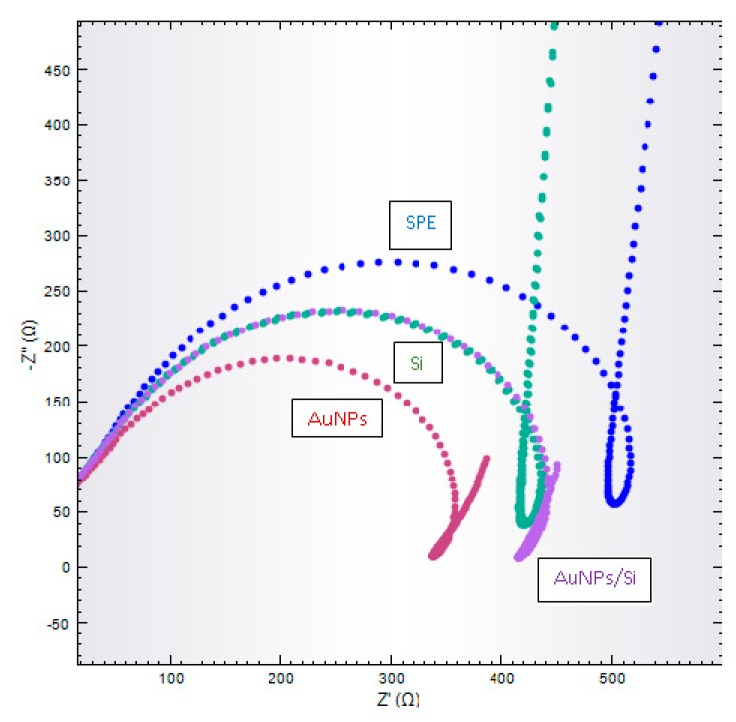
Nyquist plot for screen-printed electrodes (SPEs) (blue line), AuNPs/SPE (red line), Si/SPE (green line), and AuNPs/Si/SPE (purple line) in 0.05 M Na-phosphate buffer solution containing 0.1 M NaCl at pH7.

The 0.05 M Na-phosphate buffer with 0.1 M Na^+^ ion was used to characterize every single layer on the electrode surface. Figure 3 shows the Nyquist plots for SPE electrodes, AuNPs/SPE, HSiS/SPE, and AuNPs/HSiS/SPE. The Z’ value is the semi-circle readout from the Nyquist plot, which refers to R_CT_ and gives the electron transfer kinetic value charge of the material–electrode interface. Based on Figure 3, R_CT_ values were reduced after SPEs were doped with AuNPs. AuNPs can enhance the conductivity and reduce the charge transfer resistance (R_CT_) on the SPE electrode surface [29,30]. There was a reduction of R_CT_ values after hollow silica spheres were dropped onto the SPE electrodes due to the positively charged HSMs. As a general rule, silica is negatively charged due to the silanol group, but after the reaction with APTES, silica was positively charged with the presence of the amine group in the silica chain [5]. This was confirmed with zeta potential analysis.

**Figure 4:**
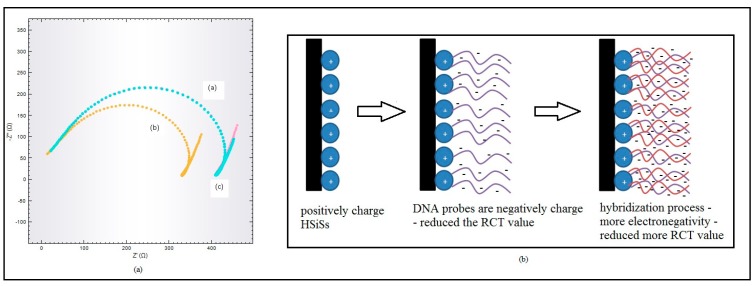
(**a**) Nyquist plot for probe DNA (blue line), target DNA (yellow line), and non-complementary DNA (pink line) in Na-phosphate buffer 0.05 M containing 0.1 M NaCl at pH 7. (**b**) Proposed interaction between silica (positive charge) and DNA (negative charge) before and after the hybridization process on the electrode surfaces.

Figure 4a shows the Nyquist plot for probe DNA, target DNA, and non-complementary DNA in Na-phosphate buffer 0.05 M containing 0.1 M NaCl at pH7. The Nyquist plots for probe DNA and non-complementary DNA are similar and proved that the hybridization process did not occur. It was found that the value of R_CT_ for the hybridized DNA with the target DNA was lower than probe DNA and non-target DNA. The negative charge on the DNA was increased due to the formation of double-stranded DNA [31,32]. DNA is a conductive biomolecule and abundant base pairs are counted as systems facilitating electron transfer to electrodes [33]. Hydrophilic double-strand DNA can partially increase electron transfer of molecules from electrolytes to electrodes, followed by infiltration through the cavity structure [32]. The interaction of HSMs and DNA is proposed in Figure 4b.

### 3.3. Effect of Buffer Solution

**Figure 5:**
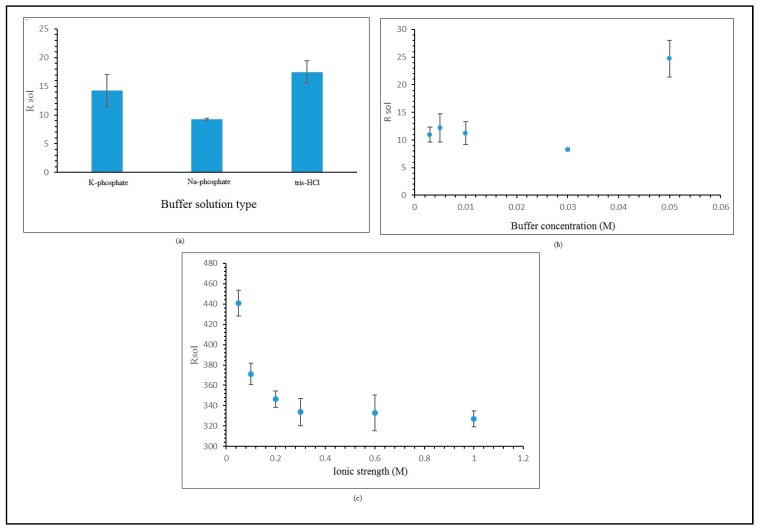
(**a**) Effect of measurement buffer solution type. (**b**) Effect of measurement buffer solution concentration. (**c**) Effect of salt concentration on measurement buffer solution.

For either electrolytes or measurement buffer solution, R_sol_ influences impedance measurement. The ionic concentration and strength in the buffer solution are crucial, and they impact the solution resistance [34]. Measurement of buffer solution resistance is not influenced by surface electrode modification [16,35]. Optimization of the measurement buffer solution is focused on several important parameters, such as the type and concentration of the buffer solution, and the effect of salt concentration on the measurement buffer solution. Optimization of the measurement buffer solution is important in the development of hybridized label-free *E. coli* DNA biosensors, as it lowers the solution resistance. The reduction of resistance in the measurement buffer solution will improve the electron transfer and subsequently intensify the stimulation of the *E. coli* DNA biosensor. Types of measurement buffer solution are studied using three types of buffer solution, as is depicted in Figure 5a, which revealed that the Na-phosphate 0.05 M at pH 7 gave the lowest resistance for the buffer solution. The Na-phosphate 0.05 M buffer solution at pH 7 eased the electron flow better than other types of buffer solution. Figure 5b,c show the effects of measurement buffer solution concentration and salt concentration, revealing that the lowest resistance value was found in a measurement buffer solution concentration of Na-phosphate 0.03 M and salt concentration of NaCl 0.3 M. All these obtained values are used in the optimization of the hybridized label-free *E. coli* DNA biosensor.

### 3.4. Optimization of Label-Free DNA Biosensor Response

All of the optimized parameters for the label-free DNA biosensor are shown in Table 1. AuNPs were used to enhance the rate of electron transfer from the surfaces of SPEs, measured with electrochemical impedance spectroscopy [29,30]. An increasing amount of AuNPs will reduce the electron charge transfer resistance rate (R_CT_), considering the electrons of AuNPs enlarge the total electrochemical active surface area of electrodes and encourage more effective electron transfer [16]. The DNA biosensor gives the lowest R_CT_ at 0.08 mg for AuNPs, and the biosensor response was maintained at 370 Ω. 

AuNPs/SPE is added with aminated hollow silica spheres (HSM) after the optimization of gold nanoparticles (AuNPs) is completed. The charge transfer resistance (R_CT_) rate will increase proportionally with the increment of the amount of gold nanoparticles due to the non-conductive silica. Aminated hollow silica microspheres (HSM) are used as the matrix for immobilization of probe DNA. According to Andrade et al. [35], the R_CT_ value depends on the insulation properties of the electrode-electrolyte interface. The considerable change of the R_CT_ value compared to other impedance components makes R_CT_ value an appropriate signal to detect the provided biosystem interface properties. As the HSM quantity increase, the DNA biosensor response increases. The hybridization rate also increases, which is depicted in the reduced R_CT_ value. Negatively charged immobilized probe DNA on the outer and inner surfaces of silica particles improve the ionic current between electrolytes and electrodes, lowering the R_CT_ value [32]. DNA hybridization with target DNA further reduces the R_CT_ value. The reduction is due to the hydrophilic properties and conformation modification of the double-stranded DNA. Double-stranded DNA that is hydrophilic can partially boost the migration of some molecules from electrolytes to electrodes, followed by infiltration through the hollow structure. After hybridization, the DNA conformation modification occurs, changing random coils of single-strand DNA into a helicoidal chain, which is more rigid [32]. The DNA biosensor produces the lowest R_CT_ value at the silica quantity of 0.04 mg. The biosensor responses start to weaken when additional aminated hollow silica is added due to agglomeration. 

The probe DNA immobilization process is divided into three steps: (1) silanization with aminosilane, (2) activation with glutaraldehyde, and (3) probe DNA covalent binding with the amine group. This hybridized label-free DNA biosensor reduces the R_CT_ signal from 0.2 to 2 µM, reducing the probe DNA capacity as a result of a higher hybridization process rate on the electrode surfaces, concurrent with probe DNA addition. The DNA hybridization rate depends on the total amount of immobilized probe DNA in the immobilization matrix [36]. The probe DNA surface density is vital, especially in a detection mechanism that relies on the intrinsic charge of the DNA phosphate backbone. Hybridization triggers elevation of negative charges on the sensor surface. This produces a potential change on the surface, which is utilized to identify hybridization on the transducer. The increment of negative charges promotes the charge transfer due to the positively charged surface electrodes. As a result, change occurs in the charge transfer resistance, which is detected using electrochemical impedance spectroscopy [37].

The presence of a DNA sequence or antibody can be confirmed by the detection of molecular bonding with its complement (selective probe), which is immobilized on the biosensor surface. The binding of target molecules with the probe causes a change in the electrical properties in surrounding electrodes. This alteration of electrical properties can be spotted by an impedance shift using impedimetric DNA or immunosensors and produces an electrical signal that is associated with affinity binding [38]. There is a slight elevation of the R_CT_ value after probe DNA is added of more than 2 µM, because when there is too much and the probe DNA is too packed, this limits the elasticity of probe DNA strands and decreases the creation rate of double-stranded DNA [39,40].

The optimization of the buffer solution for hybridization, including the type of buffer solution, effect of pH, concentration, and ionic strength, was carried out. From the experiment, 0.05 M sodium phosphate buffer solution at pH 7 was used for DNA hybridization and gave the lowest R_CT_ value compared to potassium phosphate and Tris-HCl buffer solutions. This indicates that more hybridization took place in the sodium phosphate buffer solution, resulting from a more favorable charged environment when compared with potassium phosphate and Tris-HCl buffer solutions.

The most crucial aspect in the optimization of the hybridized buffer solution is pH. Differing surrounding pH values will cause either DNA activation or DNA destruction [41]. The experiment results suggest that the hybridization rate increases from pH 5.0 to 7.0 and decreases from pH 7.5 to 8.0. DNA solubility will be reduced in an acidic surrounding, caused by the protonation of the DNA phosphodiester bond when the pH is lower than 7.0 [42]. Double-stranded DNA will be destroyed if the condition is either too acidic or too alkaline [43]. This study indicates that the most suitable pH for DNA hybridization buffer solution is pH 7.0 using sodium phosphate buffer solution, which will be used in the subsequent research.

The effects of buffer solution and ionic strength were studied using sodium phosphate buffer solution and Na^+^ ions. The pH concentration was influenced by buffer solution concentration and needed to be balanced with the salt ion concentration to ensure the kinetic reaction and chemical equilibrium were maintained [44]. The R_CT_ value decreased concurrently with the increment of sodium phosphate buffer solution concentration from 0.03 M to 0.06 M, as the buffer solution minimizes interference from any other ionic signal. Meanwhile, the R_CT_ value was increased in the range of 0.06 M to 0.07 M due to the high concentration modifying the surrounding properties of the detection mechanism and causing the sensor to become less sensitive. This study indicates that 0.06 M sodium phosphate buffer solution is the best concentration for the label-free *E. coli* DNA biosensor.

In the next experiment, the effect of ionic strength on DNA biosensor hybridization revealed that the Na^+^ ion concentration increased from 0.3 M to 0.6 M, decreasing the R_CT_ value. The value increased when the Na^+^ ion concentration increased from 0.7 M to 0.8 M. The Na^+^ ion can neutralize the negative effect of the phosphate group in the DNA phosphodiester bond [36]. The hybridization process was induced by the positive ions, which can neutralize negative charges on the probe of the DNA phosphate group [45]. The neutralization reduces the electrostatic repulsion between the DNA molecules, hence increasing the DNA interaction and leading to a more effective hybridization process. The DNA biosensor response was found to increase as the electrostatic vanished [36]. High salt content can stabilize the double-stranded DNA configuration [46,47]. For the hybridized label-free *E. coli* DNA biosensor, the optimum sodium phosphate buffer solution and Na^+^ ion concentrations are 0.06 M and 0.6 M, respectively. These values were used in the next experiments.

Initially, the effect of probe DNA immobilization time needed to be determined to find the optimum time for probe DNA to bind with hollow silica spheres. The media needed to be in equilibrium for the formation of covalent bonding [48]. The biosensor response indicates an increase of the charge transfer resistance, R_CT_, from an immobilization time of 1–3 h, and then the R_CT_ value reduces between an immobilization time of 3 h and 5 h. A slight increase of R_CT_ value occurred during the first 3 h, as only a small amount of DNA was immobilized on the surface of the electrode, leaving many electrode surfaces uncovered. In general, impedance changes are directly proportional to the surface of the analyte target under low concentration conditions [49]. 

Meanwhile, after 5 h immobilization time, there is a slightly decrease in biosensor response, with no significant change after that time. As more probe DNA is immobilized onto hollow silica microspheres, the DNA hybridization rate becomes higher, which is shown by the R_CT_ value decreasing from 3 to 5 h of immobilization time. After 5 h of HSiSs/AuNPs/SPE immersion in probe DNA, it was expected that the hollow silica particle surfaces would be entirely bound to probe DNA. Further increment of immobilization time would not influence these DNA biosensor detection abilities [50].

Based on the results of the experiment for DNA hybridization time, it was found that the optimal hybridization reaction time for the HSiSs/AuNPs/SPE-based DNA biosensor was 60 min. The R_CT_ value declines promptly with hybridization time, which clearly shows a greater DNA hybridization reaction. DNA biosensor responses increase because of the rise of duplex formations immobilized on HSiSs/AuNPs/SPE. The maximum biosensor response was achieved when the probe DNA layer hybridized thoroughly.

### 3.5. Analytical Performance of Label-Free E. coli DNA Biosensor

**Figure 6:**
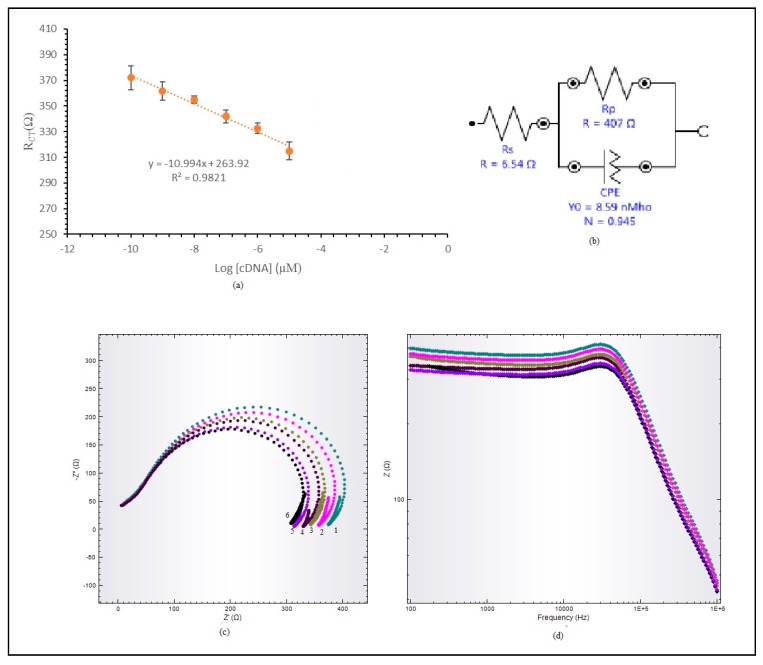
(**a**) Linear response range for label-free *E. coli* DNA biosensor. (**b**) Fitted equivalent circuit representing label-free *E. coli* DNA. (**c**) Nyquist plot. (**d**) Bode plot modulus for label-free *E. coli* DNA biosensor linear range.

The performance of the DNA biosensor was studied in sodium phosphate buffer solution at 0.06 M concentration, containing *E. coli* target DNA with differing concentrations (1 × 10^−11^–1 × 10^−3^ µM) and 0.6 M Na^+^ ions at pH 7. This was measured using the DNA biosensor technique to acquire a linear calibration curve, as shown in Figure 6. The DNA biosensor response increased when the R_CT_ value decreased. The biosensor response was almost the same at target DNA concentrations ranging from 1 × 10^−11^ to 1 × 10^−10^ µM. This DNA biosensor produces a wide linear response to target DNA with concentrations ranging from 1 × 10^−10^ to 1 × 10^−5^ µM, with a correlation coefficient of R^2^ = 0.9821, as shown in Figure 6a. A wide linear response was achieved due to hollow silica particles having inner areas that provided room for target DNA to hybridize with immobilized probe DNA. The limit of detection (LOD) for the hybridized label-free *E. coli* DNA biosensor was determined at 1.95 × 10^−15^ µM. Figure 6b shows an equivalent circuit for the label-free *E. coli* DNA biosensor. The circuit was built from a resistor, R_s_, and connected to a parallel resistance and inductor, L.

Figure 6c,d display the Nyquist plot and Bode modulus plot for the acquired linear range. The Nyquist plot in Figure 6c shows a linear response range from 1 × 10^−10^ µM to 1 × 10^−5^ µM for target DNA. X-axis and y-axis plots are marked Z’ and Z’’. This Nyquist plot consists of two parts, a semi-circle part and a linear part, each representing an electrode interface layer and electrode absorption layer. The impedance value was read from the semi-circle diameter, which is R_CT_, the reaction response of the electrochemical impedance sensor. The Bode modulus plot was plotted to observe the impedance value change, Z (ohm), against frequency. Impedance was not solely determined based on R_CT_; the frequency location during the reaction also need to be observed. Based on the Bode modulus plot in Figure 6d, the DNA frequency in sodium phosphate buffer solution is around 20 kHz. 

**Figure 7:**
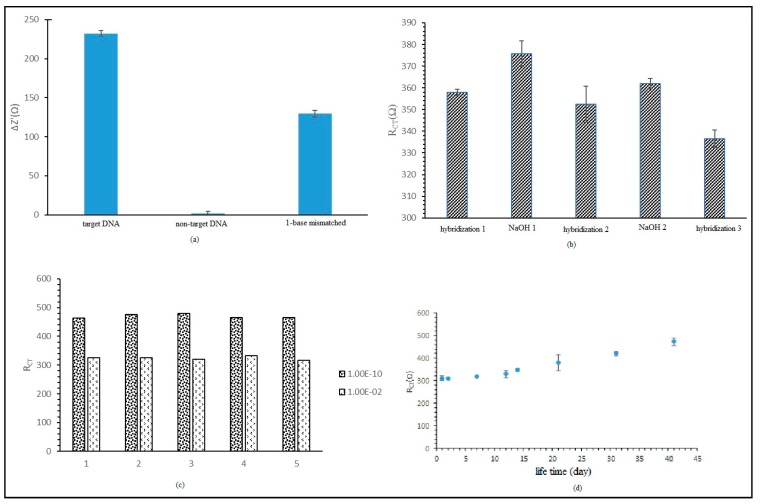
(**a**) Label-free *E. coli* DNA biosensor selectivity on target DNA, non-target DNA, and single-base mismatched DNA. (**b**) Regeneration for label-free *E. coli* DNA biosensor. (**c**) Reproducibility for label-free *E. coli* DNA biosensor. (**d**) Lifetime effect of label-free *E. coli* DNA biosensor.

The DNA biosensor response against selectivity was studied using a target DNA solution and non-target DNA solution. The DNA biosensor response showed 100% selectivity against target DNA, as displayed in Figure 7a. Negatively charged immobilized probe DNA intensifies the ionic current between electrolytes and electrodes. When hybridization with target DNA occurred, the R_CT_ value reduced because the hybridization produces double-stranded DNA, which is an attractive force between the oppositely charged increased electron transfer kinetics [20]. When probe DNA was hybridized with non-target DNA, the hybridization process did not occur and the R_CT_ value was unchanged. The R_CT_ percentage for each hybridization process with non-target DNA and 1 unpaired-base DNA strand was calculated based on the (R_CT ncDNA_/R_CT cDNA_)×*100% formula. The R_CT_ percentage for the non-target DNA was 0.84% and for the unpaired DNA base strand was 21.14% compared to target DNA R_CT_. 

The regeneration performance of the label-free *E. coli* DNA biosensor is presented in Figure 7b. From the first hybridization, we obtained an average R_CT_ value of 357.99 Ω, with a relative standard deviation (RSD) value of 1.45% (n = 3). After first immersion in NaOH, we found that the average R_CT_ value was 357.68 Ω, with a RSD value of 5.89% (n = 3). This biosensor was rehybridized with target DNA and the biosensor stimulation percentage obtained was 98.43%, with an average R_CT_ value of 352.38 Ω (RSD = 8.34% (n = 3)). Then, the biosensor was immersed again in NaOH and the R_CT_ value dropped to 361.96 Ω. The biosensor was hybridized again with target DNA and the biosensor stimulation percentage found was 94%, with average R_CT_ value of 336.55 Ω (RSD = 4.01% (n = 3)). Regeneration was stopped because it was discovered after immersion in NaOH 0.1 M that the R_CT_ value found was similar to the R_CT_ value after hybridization. It was assumed that the DNA was damaged and leached out to the matrix. Hence, this study indicated that the DNA biosensor could only be generated twice.

In addition, reproducibility and lifetime studies were conducted on the label-free *E. coli* DNA biosensor. Figure 7c shows good reproducibility, with RSD values of 7.06% and 7.81% (n = 5), as this test used two different concentrations of target DNA, 1 × 10^−10^ and 2 × 10^−2^ µM. Regarding charge transfer resistance, the R_CT_ values for both concentrations were small but could still be differentiated. The lifetime study of the label-free DNA biosensor is displayed in Figure 7d. The study indicated that the biosensor DNA response did not suggest a substantial difference for storage time up to 21 days, with R_CT_ values ranging between 2.15% and 22.46% compared to day one. For storage time from 31 to 41 days, the R_CT_ value percentage increased from 35.30% to 52.28%. The elevation of the R_CT_ value indicated a reduction of the biosensor response. This increase could be used to probe DNA or HSiSs leaching out from the electrodes. The leaching effect reduced the immobilized probe DNA amount and the rehybridization rate with target DNA. These effects will reduce the DNA biosensor response.

### 3.6. E. coli Detection from Environmental Samples

Table 2 shows label-free *E. coli* DNA biosensor that has been produced. Its effectiveness was tested with water samples from several locations. This label-free *E. coli* DNA biosensor was compared with conventional methods, namely bacterial culture on two different types of agar: coliform chromocult agar and eosin methylene blue agar. The total amount of coliform (cfu) per 100 mL for *E. coli* bacteria was calculated. The biochemical test was conducted on the bacteria formed on the agar to ensure that the bacteria was *E. coli*. ATCC 25922 *E. coli* bacteria was used in the bacterial culture and the biochemical test as control. Based on Table 2, the obtained R_CT_ value corresponded with the cfu/100 mL value for the conventional method; the R_CT_ value was low when the cfu value was high, and the R_CT_ value was high when the cfu value was low. Table 2 shows a comparison of the R_CT_ value between the electrode that was hybridized with DNA from real samples and the probe DNA HSiSs/AuNPs/SPE electrode. It was discovered that the signals for water samples from the factory and wet market had lower R_CT_ values compared to other samples. The values obtained were agreeable with the cfu value from bacteria cultured on Eosin Methylene Blue (EMB ) agar.

### 3.7. Comparison of the Analytical Performance of the Developed Label-Free DNA Biosensor with other Reported DNA Biosensors using Impedimetric Technique

The constructed label-free DNA biosensor needed to be compared with the other DNA biosensors to validate the constructed biosensor. Table 3 shows a performance comparison between the constructed biosensor with the other biosensors using impedimetric techniques.

## 4. Conclusions

In this study, a label-free DNA biosensor was successfully developed with a wide linear range and low detection limit for the determination of the *E. coli* pathogen. The label-free DNA biosensor showed a good response to the *E. coli* DNA target, which signifies that the biosensor is sensitive and has high selectivity. In the future, the developed label-free *E. coli* DNA biosensor could be used for *E. coli* pathogen detection, especially in water.

## Figures and Tables

**Table 1:** The optimum parameters for the label-free DNA biosensor.

Parameters	Optimum Amount
Amount of gold nanoparticles (AuNPs)	0.08 mg
Amount of hollow silica microspheres (HSMs)	0.04 mg
DNA probe concentration	2 µM
Types of buffer	Sodium phosphate
Sodium phosphate buffer pH	pH 7
Sodium phosphate buffer concentration	0.06 M
Ionic strength	0.6 M
DNA probe immobilization time	5 h
Hybridization time	1 h

**Table 2:** Real samples measured with conventional methods and the hybridized label-free DNA biosensor.

Sample	Biochemical Test				Impedance Signal, R_CT_	Conventional Method	
	Gramstaining	Catalase	Oxidase	Indole	(Average) (n = 3)	Chromocult Coliform Agar	Eosin Methylene Blue Agar (n = 3)
*E. coli* ATCC 25922	Negative Rod	Positive	Negative	Positive		Blue and purple colored “coliform”	Green metallic colored coliform
Water from factory surroundings	Negative Rod	Positive	Negative	Positive	343.62 Ω	Blue, purple, and red colored “coliform”	Green metallic colored coliform 75–80 cfu/100 ml
Water from wet market surroundings	Negative Rod	Positive	Negative	Positive	302.38 Ω	Blue and purple colored “coliform” (TMTC)	Green metallic colored coliform >80 cfu/100 ml
Water from café surroundings	Negative Rod	Positive	Negative	Positive	369.85 Ω	Blue, purple, and red colored “coliform”	Green metallic colored coliform 60–70 cfu/100 ml

**Table 3:** Comparison between the constructed biosensor with the other biosensors using the impedimetric technique. LOD, limit of detection.

DNA Immobilization Matrix	Virus/Bacteria	Linear Range (M)	Sensitivity (Ω/log M)	LOD (M)	R^2^	Reference
Gold nanoparticles and hollow silica particles	*E. coli*	1 × 10^−16^–1 × 10^−11^	10.994	1.95 × 10^−21^	0.9821	This study
Graphene	*E. coli*	1 × 10^−14^–1 × 10^−10^	2238.6	0.7 × 10^−15^	0.9938	[12]
Nanoporous alumina membrane	Dengue	1 × 10^−12^–1 × 10^−6^	31.75	2.7 × 10^−12^	0.96	[10]
Gold nanoparticles	Chitin enzyme gene	1.52 × 10^−10^–4.05 × 10^−8^	nA	1.0 × 10^−10^	0.9956	[51]
Gold nanoparticles and dual-layer, two-dimensional 3-mercaptopropyltrimetoxilane	oligonucleotide	1 × 10^−6^–1 × 10^−8^	nA	5 × 10^−9^	0.9962	[16]
